# The Views of Hospital Secretaries

**Published:** 1911-06-24

**Authors:** 


### THE VIEWS OF HOSPITAL SECRETARIES.

**Mr. G. P. ADAMSON, Assistant Secretary, Scarborough Hospital and Dispensary.**

With reference to the already-noted appeal for the extension of the out-patient department at Scarborough as the local memorial to King Edward, Mr. Adamson says: We shall, of course, have to take into consideration the Insurance Bill as to its probable effect on the scheme before proceeding. At the present time we are getting out elevation plans, and the various details connected with our proposed alterations, but at the same time are watching what may be the effect of the Insurance Bill.

Naturally, in a matter of this kind, opinion is divided as to its probable effect on our out-patient department. With our dispensary department in connection with the hospital we are not, in my mind, in the same position as ordinary hospitals, where the out-patient department work is necessarily smaller, as to the probable effect on our income. I quite think that in a seaside place, the same as this, where we have such a tremendous amount of casual labour, the work of our out-patient department will not be greatly diminished. As to the financial side, I am convinced that the amount we receive from the working classes in various ways will be diminished. There will be, until the time comes for the State hospitals (which to my mind is the ultimate outcome of the Insurance Bill), support from the generous public for the alleviation of the sick and needy in the treatment of both medical and surgical cases, but the whole aspect may be altered. If I were on a hospital staff as a surgeon I should most strongly object to the outside doctor getting his fee for treating a patient outside, and to my being expected to continue doing surgery in a hospital for the love of the thing—even though the societies do make, in accordance with the provisions of the Bill, grants to hospitals. I should consider my work as a surgeon as important and necessary as medical treatment.

